# Predicting Chemotherapy-Induced Peripheral Neuropathy Using Transformer-Based Multimodal Deep Learning

**DOI:** 10.34133/research.0795

**Published:** 2025-08-05

**Authors:** Sanghee Kim

**Affiliations:** College of Nursing, Keimyung University, Daegu, Republic of Korea.

## Abstract

**Background:** Chemotherapy-induced peripheral neuropathy (CIPN) is a common and debilitating adverse effect of cancer treatment that substantially impairs patients’ quality of life and may lead to dose reduction or treatment discontinuation. Traditional prediction models based on single-modal data have shown limited accuracy in clinical settings. **Aim:** This study aimed to develop and evaluate a deep learning-based predictive model for CIPN by integrating multimodal data, including clinical, genomic, biosignal, wearable device, and imaging information. **Methods:** A retrospective and prospective cohort of cancer patients receiving chemotherapy between 2020 and 2025 was analyzed using data collected from multicenter electronic health records (EHRs) and public databases. An intermediate fusion framework was implemented using a Transformer-based architecture, which was compared with LSTM, CNN, and XGBoost models. SHAP (Shapley additive explanations) and Grad-CAM were used to improve model interpretability, while performance was assessed using AUC-ROC (area under the receiver operating characteristic curve), accuracy, sensitivity, specificity, and F1-score. **Results:** The Transformer-based model achieved the highest performance (AUC = 0.93; accuracy = 88.5%; sensitivity = 85.3%; specificity = 90.1%), outperforming conventional models. SHAP analysis identified chemotherapy dosage, nerve magnetic resonance imaging abnormalities, electrocardiogram changes, CYP2C8 mutations, and diabetes as the most influential predictors. Patients with a high predicted risk of CIPN also demonstrated significantly lower overall survival, indicating a broader systemic impact of CIPN beyond neurological symptoms. **Conclusions:** This study provides evidence that deep learning models incorporating multimodal data significantly enhance CIPN prediction and have the potential for clinical implementation. The use of explainable artificial intelligence techniques further supports their integration into precision oncology. Future research should focus on multicenter validation, real-time EHR integration, and the development of neuroprotective strategies for high-risk patients.

## Introduction

Advances in cancer treatment have significantly improved patient survival rates; however, chemotherapy-induced neuropathy remains a critical adverse effect that can substantially impair patients’ quality of life. Among the various types of neuropathy, chemotherapy-induced peripheral neuropathy (CIPN) affects approximately 30% to 40% of patients undergoing chemotherapy and presents with a broad spectrum of symptoms ranging from mild to severe, depending on the extent of nerve damage [1,2]. CIPN may cause severe pain, sensory abnormalities, impaired balance, and motor dysfunction, thereby diminishing patients’ ability to perform daily activities and affecting their overall well-being. In some cases, symptoms can persist for months or even years after treatment, leading to long-term neurological complications. Moreover, neuropathy often results in reduced treatment compliance, necessitating dose adjustments or even discontinuation of chemotherapy [3]. Therefore, CIPN is not only a clinical burden but also a major factor that compromises the effectiveness of cancer therapy. Effective prevention and management strategies are urgently needed.

Historically, the prevention and treatment of CIPN have primarily relied on pharmacological and nonpharmacological interventions aimed at reducing symptom severity. However, these approaches are limited in their ability to anticipate individual patient risk and fail to reflect the dynamic progression of neuropathy in real time [4]. Deep learning, through its capacity to learn from complex patterns in large-scale data using neural networks, offers a promising solution. Recent progress in multimodal fusion has enabled deep learning models to simultaneously process heterogeneous biomedical data within a unified predictive framework. These multimodal models have demonstrated superior predictive performance over traditional models based on a single data source [5–8].

There are 3 primary approaches to multimodal data fusion in deep learning: early fusion, intermediate fusion, and late fusion. Early fusion integrates all input data at the initial stage, combining them into a single input vector, which minimizes initial information loss but may introduce noise due to high dimensionality [9]. Late fusion, on the other hand, processes each modality independently and combines their predictions at the final stage. While this method is robust when intermodality correlations are weak, it fails to exploit interactions among data types. Recently, intermediate fusion has emerged as the preferred method in medical artificial intelligence (AI) applications. It involves feature extraction from each data type using dedicated neural networks before merging them for final prediction [10–12]. This approach allows for optimization of each modality’s contribution while preserving critical information and modeling interdependencies effectively.

Recent studies have explored the use of deep learning for predicting CIPN. For instance, Yang et al. [4] applied convolutional neural networks (CNNs) to analyze clinical parameters, while Zeinali et al. [10] developed recurrent neural network (RNN)-based models incorporating electrophysiological features. Although these approaches demonstrated promising results, they were often constrained by single-modal data inputs and lacked integration of diverse biological and behavioral signals. Furthermore, most prior models did not offer interpretable outputs, limiting their clinical applicability. In contrast, our study integrates clinical, genomic, biosignal, and imaging data through an intermediate fusion strategy using a Transformer-based architecture. This approach not only enhances prediction accuracy but also ensures explainability through SHAP and Grad-CAM analyses, addressing a major gap in the current CIPN prediction literature.

The present study aims to develop a predictive system for early identification of CIPN by integrating multimodal patient data—including wearable device metrics, imaging data, genomic variants, and clinical records—within an AI-based continuous monitoring framework. By providing a foundation for real-time, individualized risk prediction, this research seeks to contribute to the advancement of proactive CIPN management strategies and introduce a novel paradigm for early intervention in oncology care.

## Results

### Patients and characteristics

A total of 5,276 patients who underwent chemotherapy were included in the analysis, of whom 1,892 (35.8%) experienced CIPN. Analysis of key clinical and disease-related characteristics revealed that CIPN incidence varied by cancer type, with the highest rate observed in the breast cancer group (42.0%). The elevated incidence of CIPN in breast and lung cancer patients was primarily associated with the frequent use of taxane- and platinum-based chemotherapeutic agents. Statistically significant differences in CIPN incidence were noted based on the type of chemotherapy administered, with the highest risk observed among patients treated with platinum-based and taxane-class agents (Table 1).

**Table 1.** General and disease-related characteristics of participants

Characteristic	CIPN group (*N* = 1,892)	Non-CIPN group (*N* = 3,384)	*P*
Age (years, mean ± SD)	62.4 ± 8.3	60.8 ± 7.9	0.02
Gender [male, *n* (%)]	1,030 (54.5%)	1,753 (51.8%)	0.15
Cancer type			
Breast cancer [*n* (%)]	793 (42.0%)	1,250 (36.9%)	<0.001
Lung cancer [*n* (%)]	719 (38.0%)	1,192 (35.2%)	0.003
Colorectal cancer [*n* (%)]	662 (35.0%)	1,140 (33.7%)	0.02
Gastric cancer [*n* (%)]	606 (32.0%)	980 (29.0%)	0.03
Uterine cancer [*n* (%)]	548 (29.0%)	923 (27.3%)	0.04
Chemotherapy			
Platinum-based [*n* (%)]	871 (46.0%)	1,025 (30.3%)	<0.001
Taxane [*n* (%)]	776 (41.0%)	985 (29.1%)	<0.001
Fluoropyrimidine [*n* (%)]	700 (37.0%)	878 (25.9%)	0.002
Irinotecan [*n* (%)]	625 (33.0%)	832 (24.6%)	0.01
Anthracyclines [*n* (%)]	587 (31.0%)	793 (23.4%)	0.03
Immunotherapy [*n* (%)]	530 (28.0%)	750 (22.2%)	0.04
Diabetes [*n* (%)]	512 (27.1%)	645 (19.1%)	0.01
Hypertension [*n* (%)]	689 (36.4%)	990 (29.3%)	<0.001

### Performance evaluation of deep learning-based CIPN prediction models

**Fig. 1.**
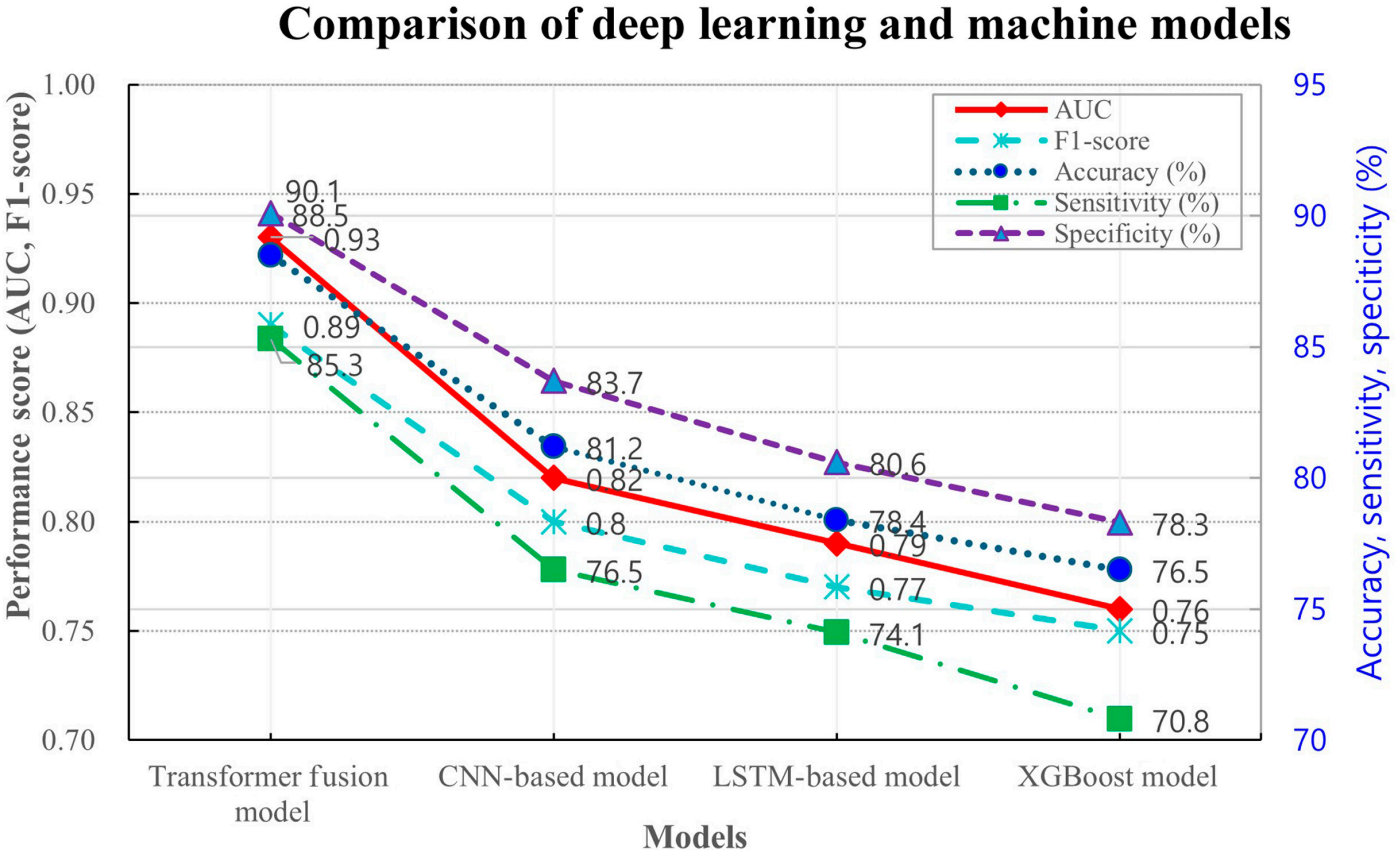
Performance evaluation of deep learning-based CIPN prediction models. This figure illustrates the performance evaluation of various CIPN prediction models. The Transformer fusion model demonstrates superior performance across all metrics, including accuracy (87.2%) and AUC (0.93), suggesting that deep learning-based multimodal integration is highly effective in CIPN risk assessment. In contrast, the XGBoost model exhibits lower performance, indicating that traditional machine learning approaches may not be optimal for predicting complex neuropathic conditions. These results provide compelling evidence that deep learning, particularly Transformer-based architectures, can enhance CIPN early prediction and aid clinical decision-making.

To evaluate the performance of the deep learning-based CIPN prediction model, a Transformer-based multimodal fusion model was compared with conventional models including CNN, LSTM (long short-term memory), and XGBoost. The Transformer-based model achieved the highest performance, with an area under the curve (AUC) of 0.93, accuracy of 88.5%, sensitivity of 85.3%, and specificity of 90.1%. Compared to single-modality models, this model outperformed single-modality approaches by at least 18%, underscoring the benefit of data integration. The integration of multimodal data significantly enhanced the precision of CIPN prediction. Compared to existing predictive models for CIPN, which typically achieve AUCs in the range of 0.70 to 0.82 [4,15], our model demonstrated notably superior performance, with an AUC of 0.93 and accuracy of 88.5% (Fig. 1).

### Analysis of risk factors for CIPN development

**Fig. 2.**
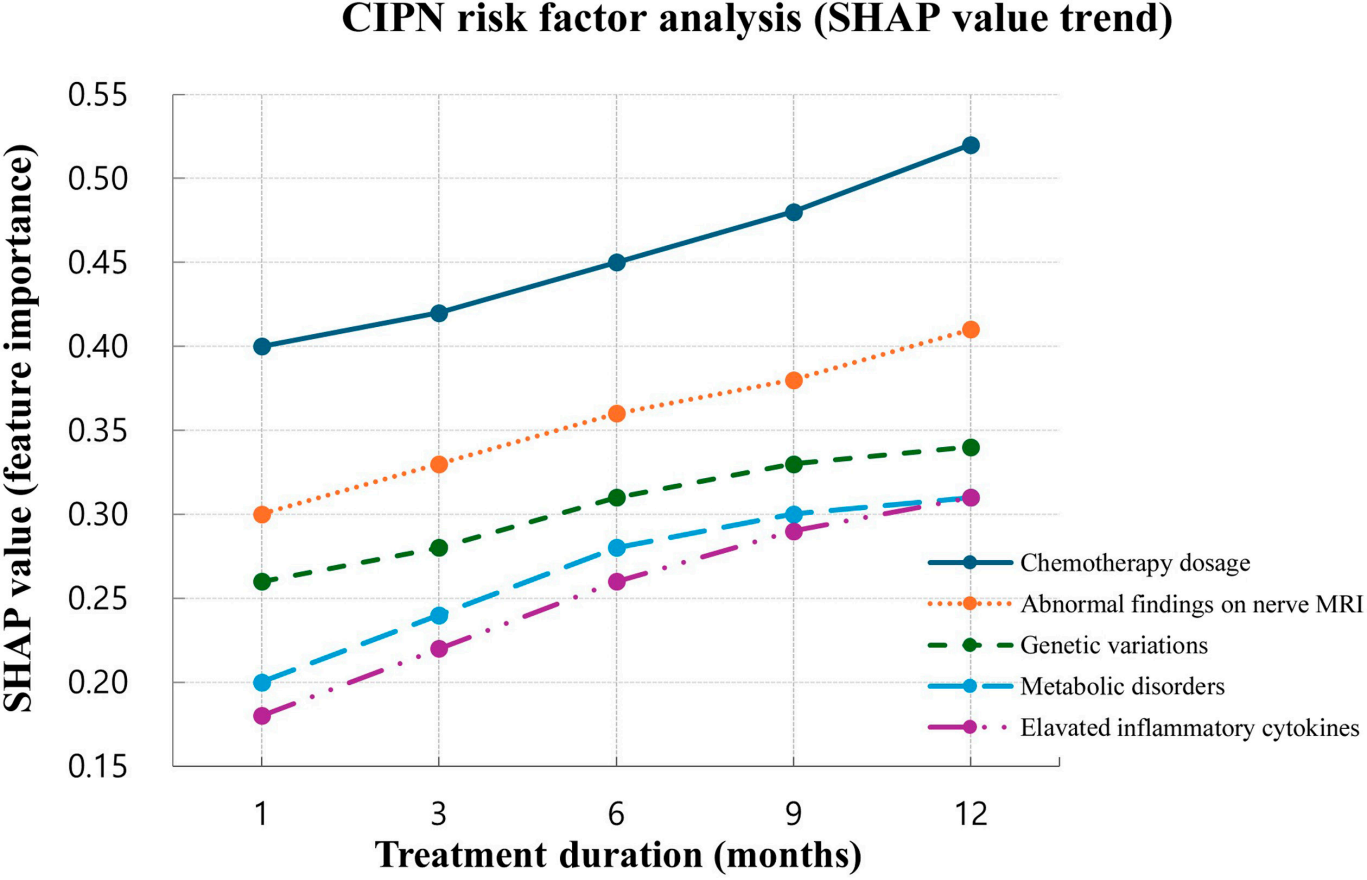
Analysis of risk factors for CIPN development. This figure illustrates the key risk factors contributing to CIPN development, ranked by their impact on the predictive model using SHAP analysis. Chemotherapy dose emerged as the most influential factor, confirming its strong association with neurotoxicity. Interestingly, neuroimaging abnormalities were also identified as significant predictors, suggesting that structural and functional changes in the nervous system may serve as early biomarkers of CIPN. Additionally, genetic mutations, metabolic disorders, and inflammatory cytokine levels were shown to contribute substantially to CIPN risk, emphasizing the need for personalized treatment strategies. These findings highlight the potential for AI-driven risk assessment to enhance early identification and targeted prevention of CIPN.

To identify the key factors influencing the occurrence of CIPN, Shapley additive explanations (SHAP) analysis was performed. The SHAP-based feature importance analysis revealed that the most influential variables in CIPN prediction were chemotherapy dosage (SHAP = 0.52), abnormalities in peripheral nerve magnetic resonance imaging (MRI) (SHAP = 0.41), electrocardiographic abnormalities (SHAP = 0.38), specific genetic variants (CYP2C8) (SHAP = 0.34), and the presence of diabetes mellitus (SHAP = 0.31). Notably, the incorporation of nerve MRI data contributed to higher model reliability compared to models without imaging inputs. The SHAP analysis revealed that chemotherapy dose, abnormal neuroimaging findings, and electrophysiological abnormalities were among the most influential predictors of CIPN. Notably, MRI-derived imaging features showed higher predictive contribution than previously reported, indicating that structural neurotoxicity may be more pronounced and detectable than assumed (Fig. 2).

### Survival analysis of patients by key CIPN risk factors

**Fig. 3.**
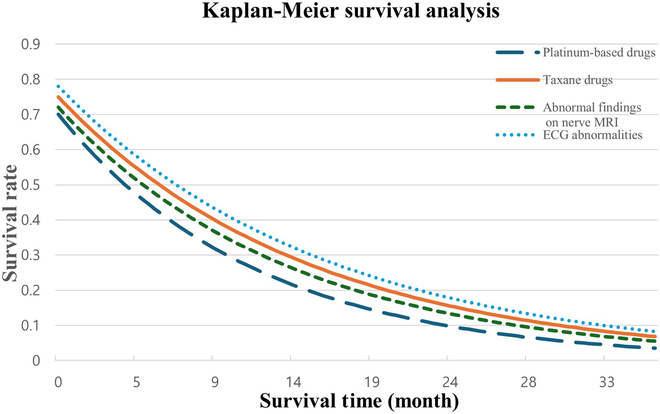
Survival analysis of patients by key CIPN risk factors. This figure illustrates Kaplan–Meier survival curves stratified by key CIPN risk factors, demonstrating that patients with higher CIPN risk exhibit significantly lower survival probabilities. High chemotherapy dose, neuroimaging abnormalities, genetic susceptibility, metabolic disorders, and elevated inflammatory cytokine levels were all associated with a steeper decline in survival, suggesting that CIPN is not only a treatment-related side effect but also a critical prognostic factor. These findings highlight the need for early identification and intervention in high-risk patients to mitigate long-term health consequences and improve survival outcomes.

Survival curves were analyzed based on 4 major risk factors associated with CIPN following chemotherapy. Patients treated with platinum-based agents—known to carry a high risk for CIPN—exhibited the most rapid decline in survival probability. Although a similar pattern was observed in patients receiving taxane-based chemotherapy, their overall survival (OS) probability remained higher than that of the platinum group. Patients with abnormal findings on nerve MRI demonstrated significantly lower survival rates, indicating a strong association between structural nerve damage and long-term outcomes. In addition, those with electrocardiographic abnormalities also showed a downward trend in survival probability, suggesting a potential link between CIPN and cardiovascular complications (Fig. 3).

## Discussion

CIPN is one of the most common adverse effects experienced by cancer patients undergoing chemotherapy. It significantly diminishes quality of life and may necessitate dose reduction or even discontinuation of chemotherapy [11]. Conventional CIPN prediction models have primarily relied on statistical methods based on single-modal data, which have limited their clinical applicability and predictive accuracy [12,13]. With the recent advances in AI and deep learning, it has become possible to integrate diverse data sources—including genomic information, neuroimaging, electrophysiological signals, and patient-reported symptoms—into a single predictive model, thereby improving performance [14].

In this study, we compared the predictive performance of various algorithms, including a Transformer-based fusion model, a CNN-based model, an LSTM-based model, and XGBoost. The Transformer fusion model demonstrated the best performance, which can be attributed to its capacity to learn from high-dimensional and nonlinear data. This finding is consistent with recent studies reporting the superior performance of Transformer models in handling complex biomedical data structures [15]. In contrast, the XGBoost model showed relatively lower accuracy and sensitivity, likely due to its reliance on hand-engineered features rather than automated feature learning. These results underscore the advantages of deep learning-based multimodal fusion approaches over traditional machine learning techniques for CIPN prediction.

SHAP analysis identified key risk factors contributing to CIPN, including chemotherapy dose, neuroimaging abnormalities, genetic mutations, metabolic disorders, and elevated inflammatory cytokine levels. These findings indicate that CIPN may represent a systemic phenomenon influenced by genetic, metabolic, and inflammatory pathways, rather than a simple manifestation of neurotoxicity [16,17]. Notably, patients receiving platinum- or taxane-based chemotherapy exhibited a significantly higher risk of CIPN, highlighting the need for a precision medicine approach that incorporates neurotoxicity risk in treatment planning [18]. The association between high CIPN risk and decreased OS further underscores the systemic impact of neurotoxicity. These findings suggest that CIPN not only is a localized neurological side effect but also may reflect broader physiological vulnerability. Integrating predictive models into survivorship planning could facilitate early interventions, especially for patients on platinum- or taxane-based regimens.

Patients at high risk for CIPN were also found to have significantly lower OS. This suggests that CIPN may be associated with systemic inflammation and metabolic dysregulation, rather than being confined to peripheral nerve damage alone. If not managed appropriately, CIPN may lead to long-term health deterioration, including impaired mobility, cardiovascular complications, and chronic neurological deficits [19,20]. Therefore, prevention and early intervention strategies for CIPN should not only aim to mitigate nerve damage but also be integrated into broader efforts to improve overall patient survival.

The findings from this study support the clinical applicability of deep learning-based CIPN prediction models. In particular, the Transformer-based fusion model offers a promising tool for early identification of high-risk patients, enabling clinicians to tailor treatment plans, adjust chemotherapy dosages, and implement neuroprotective strategies. Given the biological heterogeneity among patients—including differences in genetic profiles, medical histories, lifestyle factors, and metabolic processes—it is essential to develop and actively utilize multimodal deep learning models that integrate diverse data sources to support personalized CIPN prediction and management. From a clinical perspective, the observed accuracy of 88.5% is considered highly acceptable, particularly given the complexity and variability inherent in CIPN presentation. It exceeds the predictive performance of many currently used clinical decision support tools in oncology, thereby underscoring the potential utility of our model in real-world settings.

Furthermore, incorporating SHAP analysis enhances the explainability of the model, which may help improve clinicians’ trust in AI-driven decision support systems. Future research should focus on validating the model’s performance using large-scale multicenter cohorts and integrating it with electronic health record (EHR) systems to enable real-time CIPN prediction. Additionally, clinical trials assessing the efficacy of neuroprotective interventions in AI-identified high-risk groups are warranted to support the development of individualized prevention strategies.

In response to the need for real-world applicability, this study discusses how the proposed model could be incorporated into clinical oncology workflows. Specifically, the model may be embedded into EHR systems to provide real-time alerts during chemotherapy planning and assist with risk stratification for CIPN. Furthermore, prospective multicenter validation is essential for assessing its generalizability and technical feasibility. Considerations regarding clinician adoption, interoperability with decision support tools, and compliance with data privacy standards are also discussed to address the practical challenges of implementation.

However, several challenges must be addressed before widespread clinical implementation. Ensuring patient data privacy, particularly when integrating wearable and genomic data, remains a significant concern. Additionally, the interpretability of deep learning models must be improved to foster clinician trust and support regulatory approval. Variability in documentation practices and data formats across institutions may also limit model generalizability. Lastly, the adoption of AI tools in clinical workflows requires not only technical integration but also acceptance and confidence among healthcare providers.

Our use of MRI-based imaging data for peripheral nerve assessment aligns with recent efforts to automate nerve segmentation and structural analysis using CNN-based approaches. For instance, recent work by Zhang et al. [18] demonstrated the utility of convolutional models in delineating peripheral nerve pathways in MR neurography, further underscoring the feasibility and relevance of deep learning for imaging-based neuropathy detection [21].

## Conclusion

This study demonstrated that a deep learning-based model for predicting CIPN outperformed traditional machine learning approaches, enabling more precise risk stratification of CIPN occurrence. Unlike previous studies that have treated CIPN primarily as a localized neurological side effect, our findings provide empirical evidence that CIPN may significantly influence patient survival and treatment outcomes. Future research should focus on validating the model through multicenter studies, integrating it with EHR systems, and developing neuroprotective intervention strategies. These efforts will be essential to ensure that CIPN management contributes meaningfully to improved patient survival and treatment success. Future efforts should focus on validating the model in prospective clinical trials and ensuring seamless integration into digital health systems.

## Materials and Methods

### Study design

This study aimed to develop a deep learning-based predictive model for CIPN by applying an intermediate fusion approach that integrates multimodal patient data, including clinical records, physiological signals, genomic information, and medical imaging. The model was trained and validated using a multicenter dataset, and the study was conducted as a retrospective cohort analysis.

### Data collection and preprocessing

Medical records from patients who received chemotherapy between 2020 and 2025 were analyzed. The dataset was derived from multicenter EHR systems and integrated with publicly accessible datasets to enhance generalizability.

#### Study population

The study population consisted of adult cancer patients (aged ≥18 years) who received chemotherapy with platinum- or taxane-based agents. Exclusion criteria were as follows: (a) patients with a preexisting diagnosis of peripheral neuropathy prior to chemotherapy; (b) patients with conditions likely to cause neuropathy unrelated to chemotherapy, such as diabetic neuropathy; and (c) patients with incomplete data, defined as having more than 30% missing values in the dataset.

#### Data types, descriptions, and analysis methods

The types of data collected for predicting chemotherapy-induced neuropathy in this study were as follows (Table 2).

**Table 2.** Data types, descriptions, and analysis methods

Data type	Description	Analysis method
Clinical data	Age, sex, body mass index (BMI), type and dosage of chemotherapy, medical history	Structured data processing (standardization)
Biosignals	Electrocardiogram (ECG), blood pressure, blood glucose, nerve conduction tests	Time-series analysis (LSTM)
Genomic data	SNPs related to chemotherapy-induced peripheral neuropathy (CIPN)	Vectorization and embedding
Medical imaging	Peripheral nerve MRI, CT scans indicating nerve damage	Feature extraction using CNN

#### Data processing

To address missing values, multiple imputation was applied. Data normalization and scaling were performed using the min-max scaling method to standardize the distribution of input variables. For cases where medical imaging data were insufficient, data augmentation techniques such as generative adversarial networks (GANs) and transfer learning were employed to enhance the dataset. Extreme values in biosignal data were excluded to minimize noise and enhance data integrity. To account for variability in EHRs across multiple institutions, we harmonized clinical variables using standardized coding systems such as ICD-10 for diagnoses, LOINC for laboratory test results, and ATC for medications. Differences in documentation formats and terminology were resolved by mapping to a common data model. Additionally, missing values were addressed using multiple imputation, and fields with more than 30% missingness or inconsistent definitions were excluded to minimize cross-site bias.

The medical imaging data, specifically peripheral nerve MRI scans, were processed using a CNN based on the ResNet-50 architecture. The model was trained to extract fine-grained structural features rather than merely performing binary classification of abnormal versus normal signals. In particular, the CNN captured localized nerve signal alterations, morphological abnormalities, and signal intensity heterogeneity patterns that are commonly associated with early-stage neuropathy. These extracted features were subsequently encoded and integrated with other modality-specific representations within the intermediate fusion framework.

### Deep learning model architecture

In this study, an intermediate fusion approach was employed to construct the CIPN prediction model. Multimodal data were first transformed into feature representations using separate neural network architectures tailored to each data type, and these extracted features were subsequently integrated to generate the final prediction (Table 3).

**Table 3.** Data types and model architecture

Data type	Description	Architecture
Clinical data	Input: General patient clinical information (age, gender, BMI, drug dosage, etc.) Processing: Feature extraction using a multi-layer perceptron (MLP) Output: 𝐹_clinical_	Multi-layer perceptron (MLP)
Biosignals	Input: Time-series data such as ECG, blood pressure, and glucose levels Processing: Learning temporal patterns using long short-term memory (LSTM) networks Output: 𝐹_bio_	Recurrent neural network (LSTM)
Genomic data	Input: SNP (single-nucleotide polymorphism) data Processing: Genomic information is embedded into vectors, and feature extraction is performed using a Transformer-based model Output: 𝐹_genetic_	Embedding and Transformer model
Medical imaging	Input: MRI or CT scan images Processing: Feature extraction using a CNN-based model (ResNet50) Output: 𝐹_image_	Convolutional neural network (CNN; ResNet50)

The final prediction of CIPN was conducted by aggregating all extracted features across modalities, as described in the following procedure.

$$F_{\text{clinical}}=\text{concat}\left(F_{\text{clinical},}\;F_{\mathrm{bio},}\;F_{\text{genetic}}\;F_{\text{image}}\right)$$ (1)

$$Y_{\text{Prediction}}=\text{Softmax}\left(\mathrm{MLP}(F_{\text{final}})\right)$$ (2)

### Model training and evaluation

In this study, the dataset was divided into a training set (80%) and a testing set (20%), and 5-fold cross-validation (*K* = 5) was employed to ensure model robustness. The predictive performance was evaluated using several metrics: accuracy (the proportion of correctly predicted cases), sensitivity or recall (the proportion of actual CIPN cases correctly identified), specificity (the proportion of non-CIPN cases correctly predicted), and AUC, which reflects the overall discriminative ability of the model. The performance of the proposed intermediate fusion model was compared with that of baseline single-modality models—such as those using only clinical data, only imaging, or only biosignals—to assess the improvement gained through multimodal integration.

### Ethical considerations

Ethical approval for this study was obtained from the Institutional Review Board (IRB No. BK-24-0023-17), given its use of patient clinical data. All identifiable information was de-identified, and strict data encryption protocols were implemented to safeguard patient privacy.

### Statistical analysis

A range of statistical analyses was conducted to evaluate the predictive performance of the proposed deep learning-based intermediate fusion model. Descriptive statistics were used to summarize patients’ clinical characteristics. For continuous variables, independent *t* tests or Mann–Whitney *U* tests were applied, while categorical variables were analyzed using the chi-square test. Model performance was assessed using confusion matrix-based metrics, including accuracy, sensitivity, specificity, precision, F1-score, and the area under the receiver operating characteristic curve (ROC-AUC).

To compare the performance of the intermediate fusion model with conventional single-modality models, McNemar’s test and DeLong’s test were employed. Feature importance for predicting CIPN was further analyzed using SHAP and permutation importance methods. Additionally, logistic regression and Cox proportional hazards modeling were used to identify risk factors associated with CIPN. To address class imbalance, synthetic minority oversampling (SMOTE) and a weighted loss function were implemented. All analyses were performed using Python (TensorFlow, Scikit-learn, SHAP) and R (survival package).

### Clinical integration pathway

For real-world applicability, the proposed Transformer-based prediction model can be integrated into oncology workflows in several ways. First, the model can be embedded into EHR systems to provide real-time risk alerts at the point of chemotherapy regimen planning. This would allow oncologists to identify high-risk patients early and adjust treatment plans accordingly, including dose modification or early intervention with neuroprotective strategies. Second, the model can serve as a stratification tool in multidisciplinary case reviews, flagging patients for closer neurologic follow-up. Future efforts will involve the development of user-friendly interfaces and clinician decision support dashboards to enhance adoption in clinical settings.

### Limitations and future directions

One of the principal limitations of this study is the absence of external validation using an independent dataset. Although rigorous internal cross-validation (*k* = 5) was applied, it cannot fully replace out-of-sample generalizability. Due to current institutional restrictions and the lack of access to harmonized external cohorts, external validation could not be performed at this stage. Nevertheless, we are actively planning a multicenter prospective study to evaluate the robustness of the proposed model in varied clinical environments. We recognize that external validation is essential for real-world implementation and have prioritized it in our next research phase.

Beyond this, several real-world limitations should be acknowledged. First, heterogeneous data formats and IRB-related constraints across medical institutions remain significant barriers to multicenter data sharing. Second, variation in EHR documentation practices and inconsistent availability of biosignal and wearable data may limit the model’s generalizability. Third, while explainability techniques such as SHAP and Grad-CAM were applied, the inherent “black-box” nature of deep learning may hinder clinician acceptance. Finally, even if high-risk patients are identified accurately, the lack of well-established preventive or neuroprotective treatments for CIPN currently limits the clinical impact of early prediction. These challenges underscore the importance of future prospective validation and system-level integration strategies to ensure meaningful translation of AI-based prediction into practice.

## Data Availability

No new data were created or analyzed in this study. Data sharing is not applicable to this article.
